# Supplementary material for: Genetic and pharmacological relationship between P-glycoprotein and increased cardiovascular risk associated with clarithromycin prescription: An epidemiological and genomic population-based cohort study in Scotland, UK
Source: PLoS Med. 2020 Nov 23;17(11):e1003372. doi: 10.1371/journal.pmed.1003372 (PMC7682888; doi:10.1371/journal.pmed.1003372)
Supplement: S1 Checklist — STROBE, Strengthening the Reporting of Observational Studies in Epidemiology. (DOCX) [file pmed.1003372.s001.docx]

STROBE Statement—checklist of items that should be included in reports of observational studies

|  | | Item No. | Recommendation | Section/Paragraph Number | | | Relevant text from manuscript | |
| --- | --- | --- | --- | --- | --- | --- | --- | --- |
| **Title and abstract** | | 1 | (*a*) Indicate the study’s design with a commonly used term in the title or the abstract | Title | | | An Epidemiological and Genomic Population-Based Cohort Study | |
|  |  |  | (*b*) Provide in the abstract an informative and balanced summary of what was done and what was found | Abstract, Methods and Findings | | | We conducted an observational cohort study of patients prescribed clarithromycin or amoxicillin in the community in Tayside, Scotland (population ~400,000) between 2004 and 2014 and a genomic observational cohort study evaluating genotyped patients from the GoDARTS study, a longitudinal cohort study of 18,306 individuals with and without type 2 diabetes recruited between 1989 and 2015. Two single nucleotide polymorphisms associated with P-gp activity were evaluated (rs1045642 and rs1128503 – AA genotype associated with lowest P-gp activity). The primary outcome for both analyses was CV hospitalisation following prescription of clarithromycin versus amoxicillin at 0-14 days, 15-30 days and 30 days to 1 year. In the observational cohort study we calculated hazard ratios adjusted for likelihood of receiving clarithromycin using inverse proportion of treatment weighting as a covariate, while in the pharmacogenomic study hazard ratios were adjusted for age, sex, history of myocardial infarction and history of COPD.  The observational cohort study included 48,026 individuals with 202,761 discrete antibiotic prescribing episodes (33,759 clarithromycin – mean age 73 years, 42% male; 169,002 amoxicillin – mean age 74 years, 45% male). Clarithromycin use was significantly associated with increased risk of CV hospitalisation compared to amoxicillin at both 0-14 days (HR 1.31; 95% CI 1.17-1.46, p<0.001) and 30 days-1 year (HR 1.13; 95% CI 1.06-1.19, p<0.001), with the association at 0-14 days modified by use of P-gp inhibitors or substrates (interaction p value 0.029). In the pharmacogenomic study (13,544 individuals with 44,618 discrete prescribing episodes, [37,497 amoxicillin – mean age 63 years, 56% male; 7,121 clarithromycin – mean age 66 years, 47% male]), when prescribed clarithromycin, individuals with genetically-determined lower P-gp activity had a significantly increased risk of CV hospitalisation at 30 days to 1 year compared to heterozygotes or those homozygous for the non-P-gp lowering allele (rs1045642 AA: HR 1.39, 95% CI 1.20-1.60, GG/GA: HR 0.99, 95% CI 0.89-1.10, p=0.85, interaction p value <0.001 and rs1128503 AA 1.41, 95% CI 1.18-1.70, p<0.001, GG/GA: HR 1.04, 95% CI 0.95-1.14, p=0.43, interaction p value <0.001). The main limitation of our study is its observational nature, meaning that we are unable to definitively determine causality. | |
| Introduction | | | | | |  | |  |
| Background/rationale | | 2 | Explain the scientific background and rationale for the investigation being reported | Introduction, paragraph 1 | | | Clarithromycin is a widely prescribed macrolide antibiotic, comprising around 15% of all antibiotic prescriptions in primary care in the United Kingdom, that is recommended for treatment of patients with lower respiratory tract infections either as monotherapy or in combination.1-3 However, there has been growing concern regarding increased cardiovascular (CV) risk of clarithromycin. | |
| Objectives | | 3 | State specific objectives, including any prespecified hypotheses | Introduction, paragraph 4 | | | We hypothesised that the increased cardiovascular risk with clarithromycin may be associated with concurrent use of P-gp inhibitors, and that individuals with genotypes associated with low P-gp activity, a proxy for P-gp inhibition, would also have an increased cardiovascular risk when prescribed clarithromycin. | |
| Methods | | | | | |  | |  |
| Study design | | 4 | Present key elements of study design early in the paper | Methods, paragraph 1 | | | The study consisted to two parts – a traditional observational cohort study; and a pharmacogenomic study. | |
| Setting | | 5 | Describe the setting, locations, and relevant dates, including periods of recruitment, exposure, follow-up, and data collection | Methods, paragraph 2 | | | The study population was all ∼400,000 residents of the Tayside region of Scotland registered with an NHS Tayside general practice at any point in the study period (2004–14). | |
| Participants | | 6 | (*a*) *Cohort study*—Give the eligibility criteria, and the sources and methods of selection of participants. Describe methods of follow-up  *Case-control study*—Give the eligibility criteria, and the sources and methods of case ascertainment and control selection. Give the rationale for the choice of cases and controls  *Cross-sectional study*—Give the eligibility criteria, and the sources and methods of selection of participants | Methods, paragraphs 2 and 3 | | | Demographic and community prescribing data were obtained through the Health Informatics Centre (HIC), University of Dundee, which provides anonymised linked individual patient data including prescribing of antibiotics as previously described.23 These datasets were linked to other datasets including demographic, clinical, hospital admission and mortality data that are linked by a unique 10-digit patient identifier (the Community Health Index number) that is used for all health care activities in Scotland.  Cohort Study: Prescribing data between 2004 and 2014 was used to identify all patients over 18 years old who were prescribed clarithromycin (alone or in combination with amoxicillin or another antibiotic) over this period. A control group of individuals prescribed amoxicillin as a sole antibiotic was also identified.  Pharmacogenomic Study: A blood sample for genotyping was obtained from individuals at baseline and all patients consented to electronic record linkage allowing details on prescriptions from 1989 to present and outcome data on deaths and hospitalisations. In this part of the study, we again only included individuals who had received a prescription for either amoxicillin or clarithromycin. | |
|  |  |  | (*b*) *Cohort study*—For matched studies, give matching criteria and number of exposed and unexposed  *Case-control study*—For matched studies, give matching criteria and the number of controls per case |  | | | n/a | |
| Variables | | 7 | Clearly define all outcomes, exposures, predictors, potential confounders, and effect modifiers. Give diagnostic criteria, if applicable | Methods, paragraph 6 (Statistical Analysis) | | | Adjusted hazard ratios in the observational study are reported using the inverse probability treatment weighting (IPTW), using the propensity score for likelihood of prescription of clarithromycin based on baseline variables reported in Table 1 [33]. Analysis for the pharmacogenomic study was informed by the design and results of the observational study. In the pharmacogenomic study, a multivariable Cox regression analysis was performed for the association between clarithromycin use versus amoxicillin on CV hospitalisation with adjustment for age at the time of antibiotic prescription, sex, history of prior MI, chronic obstructive pulmonary disease (COPD) and type 2 diabetes. | |
| Data sources/ measurement | | 8* | For each variable of interest, give sources of data and details of methods of assessment (measurement). Describe comparability of assessment methods if there is more than one group | Methods, paragraphs 2 and 3 | | | As per item 6 | |
| Bias | | 9 | Describe any efforts to address potential sources of bias | Methods - Statistical analysis. | | | Inverse probability treatment weighting, use of genomics | |
| Study size | | 10 | Explain how the study size was arrived at | Methods, paragraphs 2-4 | | | Observational Cohort: Prescribing data between 2004 and 2014 was used to identify all patients over 18 years old who were prescribed clarithromycin (alone or in combination with amoxicillin or another antibiotic) over this period. A control group of individuals prescribed amoxicillin as a sole antibiotic was also identified.  Pharmacogenomic Study: A blood sample for genotyping was obtained from individuals at baseline and all patients consented to electronic record linkage allowing details on prescriptions from 1989 to present and outcome data on deaths and hospitalisations. In this part of the study, we again only included individuals who had received a prescription for either amoxicillin or clarithromycin. Collection and analysis of data in GoDARTS was approved by the East of Scotland Research and Ethics Committee. All participants had given written consent for their data to be linked and analysed for research purposes.  We selected 2 single-nucleotide polymorphisms (SNPs) within the human multidrug‐resistance MDR1 gene (ABCB1), which codes for P-gp, which have been shown to be associated with P-glycoprotein activity in white healthy volunteers and for which there was a reasonably high prevalence of each genotype – rs1045642, rs1128503. | |
| Quantitative variables | 11 | Explain how quantitative variables were handled in the analyses. If applicable, describe which groupings were chosen and why | | | Methods, statistical analysis paragraph 1 | Continuous variables are reported as mean ± standard deviation and categorical variables are reported as number and percentage. | |  |
| Statistical methods | 12 | (*a*) Describe all statistical methods, including those used to control for confounding | | | Statistical Analysis | Standardised mean differences (SMD) between the clarithromycin and amoxicillin groups are reported, with an SMD >0.1 considered a significant difference between the groups.  In the observational cohort study Cox proportional hazards regression was performed for the outcome of CV hospitalisation or hospitalisation for MI at 0-14 days, 15-30 days and 30 days to 1 year. Additionally, we evaluated the endpoints of all-cause and CV mortality. Adjusted hazard ratios in the observational study are reported using the inverse probability treatment weighting (IPTW), using the propensity score for likelihood of prescription of clarithromycin based on baseline variables reported in Table 1 [33]. Analysis for the pharmacogenomic study was informed by the design and results of the observational study. In the pharmacogenomic study, a multivariable Cox regression analysis was performed for the association between clarithromycin use versus amoxicillin on CV hospitalisation with adjustment for age at the time of antibiotic prescription, sex, history of prior MI, chronic obstructive pulmonary disease (COPD) and type 2 diabetes. Interaction testing was performed to determine whether there was a significant difference between amoxicillin and clarithromycin prescribing depending on P-gp activity. All tests were two-sided and a p value <0.05 was considered statistically significant. Statistical analysis was performed using SAS version 9.4 and R version 3.5.1. | |  |
|  |  | (*b*) Describe any methods used to examine subgroups and interactions | | | Statistical Analysis | Interaction testing was performed to determine whether there was a significant difference between amoxicillin and clarithromycin prescribing depending on P-gp activity. | |  |
|  |  | (*c*) Explain how missing data were addressed | | |  | n/a | |  |
|  |  | (*d*) *Cohort study*—If applicable, explain how loss to follow-up was addressed  *Case-control study*—If applicable, explain how matching of cases and controls was addressed  *Cross-sectional study*—If applicable, describe analytical methods taking account of sampling strategy | | |  | n/a | |  |
|  |  | (*e*) Describe any sensitivity analyses | | |  | n/a | |  |
| Results | | | | | | | |  |
| Participants | 13* | (a) Report numbers of individuals at each stage of study—eg numbers potentially eligible, examined for eligibility, confirmed eligible, included in the study, completing follow-up, and analysed | | | Results, paragraphs 1 and 4 | Over the duration of the study there were 33,759 prescriptions for clarithromycin for 11,489 unique individuals and 169,002 amoxicillin prescriptions for 36,537 unique individuals.; In total there were 42,272 amoxicillin prescriptions from 8,513 unique individuals and 7,135 clarithromycin prescriptions from 5,031 unique individuals. | |  |
|  |  | (b) Give reasons for non-participation at each stage | | |  | n/a | |  |
|  |  | (c) Consider use of a flow diagram | | |  | n/a | |  |
| Descriptive data | 14* | (a) Give characteristics of study participants (eg demographic, clinical, social) and information on exposures and potential confounders | | | Results, paragraphs 1 and 4 | The mean age at prescription was 73.3 ± 12.3 years in the clarithromycin group and 41.9% were male while in the amoxicillin group the mean age was 74.2 ± 13.2 years and 44.7% were male.;  Mean age at prescription was lower in the amoxicillin cohort (63.6 ± 13.4 vs. 65.9 ± 12.0 years, p<0.001). There was a higher percentage of females prescribed amoxicillin than clarithromycin (54.6% vs. 53.0%, p<0.001); Table 1 | |  |
|  |  | (b) Indicate number of participants with missing data for each variable of interest | | |  | n/a | |  |
|  |  | (c) *Cohort study*—Summarise follow-up time (eg, average and total amount) | | |  | n/a (decided by outcome up to 1 year) | |  |
| Outcome data | 15* | *Cohort study*—Report numbers of outcome events or summary measures over time | | | Results, paragraphs 2 and 6 | Within the first 14 days following prescription of clarithromycin there were 559 CV hospitalisations (1.6% of all prescriptions) and 289 deaths from any cause, compared to the amoxicillin group in which there were 2,355 CV hospitalisations (1.2% of all prescriptions) and 1,601 deaths from any cause.;  In total there were 953 CV hospitalisations within 1 year of antibiotic prescription in the clarithromycin group (13.3% of all clarithromycin prescriptions) compared to 3,160 (7.5%) in the amoxicillin group. There were 359 (5.0%) hospitalisations for MI within 1 year of prescription in patients prescribed clarithromycin compared to 795 (1.9%) in those prescribed amoxicillin. | |  |
|  |  | *Case-control study—*Report numbers in each exposure category, or summary measures of exposure | | |  |  | |  |
|  |  | *Cross-sectional study—*Report numbers of outcome events or summary measures | | |  |  | |  |
| Main results | 16 | (*a*) Give unadjusted estimates and, if applicable, confounder-adjusted estimates and their precision (eg, 95% confidence interval). Make clear which confounders were adjusted for and why they were included | | | Results | Throughout results section | |  |
|  |  | (*b*) Report category boundaries when continuous variables were categorized | | | Results | Throughout results section | |  |
|  |  | (*c*) If relevant, consider translating estimates of relative risk into absolute risk for a meaningful time period | | |  |  | |  |
| Other analyses | 17 | Report other analyses done—eg analyses of subgroups and interactions, and sensitivity analyses | | | Figures | Figures 1 and 2 | |  |
| Discussion | | | | | | | |  |
| Key results | 18 | Summarise key results with reference to study objectives | | | Discussion, paragraph 1 | Our study has two key findings. First, not only was clarithromycin use associated with increased CV risk compared to amoxicillin, as other studies have reported, but we have identified that the risk is particularly increased in those taking P-glycoprotein inhibitors concurrently. This finding was strengthened by use of propensity-score weighting, and a similar finding was observed in the observational analysis of the genomic cohort. Second, in order to strengthen our findings, we have shown in a genomic study, which should reduce the risk of confounding by indication, that the association of clarithromycin with CV hospitalization between 30 days and 1 year was significantly increased in individuals with lower genetically-predicted levels of P-gp activity. | |  |
| Limitations | 19 | Discuss limitations of the study, taking into account sources of potential bias or imprecision. Discuss both direction and magnitude of any potential bias | | | Discussion, “Strengths and Limitations” | “Our study has some limitations. First, there are inherent limitations with any observational study, although our use of propensity weighting for likelihood of prescription and genomics do obviate some of these…” | |  |
| Interpretation | 20 | Give a cautious overall interpretation of results considering objectives, limitations, multiplicity of analyses, results from similar studies, and other relevant evidence | | | Conclusion | “We found that clarithromycin use was associated with an increased risk of CV hospitalisation up to 1 year post prescription compared to amoxicillin. There appears to be an effect modification via P-glycoprotein, with a particularly increased risk of adverse CV events with clarithromycin in patients also taking drugs that are P-glycoprotein substrates or those with lower genetically-predicted levels of P-glycoprotein activity.” | |  |
| Generalisability | 21 | Discuss the generalisability (external validity) of the study results | | | Discussion, “What this study adds to existing research” | Comparison with other studies “Concerns regarding the CV risk of macrolide antibiotics have been present for several years(37), and were strengthened by the results of the CLARICOR randomised trial, which contrary to the authors’ original hypothesis, demonstrated an increased risk of CV mortality at both 3 and 10 years.(4, 16) These results have been further supported by several large observational studies(5-7, 38) and meta-analyses(9, 10), which have reported increased CV risk of myocardial infarction and CV hospitalisation up to 1 year after macrolide prescription. Other macrolides such as azithromycin have also been linked with increased CV risk.(8)…” | |  |
| Other information | |  | | | | | |  |
| Funding | 22 | Give the source of funding and the role of the funders for the present study and, if applicable, for the original study on which the present article is based | | | “Role of the Funding Source” | The funder of the study had no role in study design, data collection, data analysis, data interpretation, or writing of the report. The corresponding author had full access to all the data in the study, and the corresponding author had final responsibility for the decision to submit for publication. | |  |

*Give information separately for cases and controls in case-control studies and, if applicable, for exposed and unexposed groups in cohort and cross-sectional studies.

**Note:** An Explanation and Elaboration article discusses each checklist item and gives methodological background and published examples of transparent reporting. The STROBE checklist is best used in conjunction with this article (freely available on the Web sites of PLoS Medicine at http://www.plosmedicine.org/, Annals of Internal Medicine at http://www.annals.org/, and Epidemiology at http://www.epidem.com/). Information on the STROBE Initiative is available at www.strobe-statement.org.
