# Supplementary material for: Genetic and pharmacological relationship between P-glycoprotein and increased cardiovascular risk associated with clarithromycin prescription: An epidemiological and genomic population-based cohort study in Scotland, UK
Source: PLoS Med. 2020 Nov 23;17(11):e1003372. doi: 10.1371/journal.pmed.1003372 (PMC7682888; doi:10.1371/journal.pmed.1003372)
Supplement: S1 Text — (DOCX) [file pmed.1003372.s002.docx]

**INITIAL ANALYSIS PLAN AS PER ORIGINAL FUNDING PROPOSAL (02 Nov 2016)**

**Study Design and Patient Selection:** We plan to conduct a retrospective data analysis study. In collaboration with the Health Informatics Centre (HIC) we will use record linkage to identify patients within Tayside who have been prescribed macrolide antibiotics either in the community or during a hospital admission. A control cohort will be patients prescribed penicillin-based antibiotics such as amoxicillin alone.

**Protocol and Measurements of Interest**

After identifying the cohort of patients who have taken macrolide antibiotics using record linkage, we will determine the risk of CV death in patients on macrolides vs. those on penicillins alone. Assuming the risk of CV death is higher in patients taking macrolide antibiotics, we will then divide the cohort into two. We will develop a validation cohort using patients resident in Dundee postcodes (DDx). Based on baseline demographic and clinical features, we will aim to develop a risk score for prediction of CV mortality in patients taking clarithromycin using a Cox proportional hazards statistical model. We will then validate this risk prediction model in patients resident in Perth postcodes (PHx).

**Statistical Analysis**

Results will be analysed using a standard statistics package (e.g. SPSS). Comparisons between groups will be performed using T-tests and chi-square tests as appropriate. Assessment of mortality and development of the risk prediction model will be performed using a Cox proportional hazards model, adjusting for relevant confounding variables.

Using this model we will identify whether patients taking macrolides are at increased risk of CV mortality compared to amoxicillin use in a community based cohort (most studies to date have looked at hospitalised patients). We will then identify co-variates that are significantly and independently associated with cardiovascular events and mortality in clarithromycin users in order to derive a risk score.

Beta-coefficients from the model will be used to generate appropriate weighting of risk factors and predictive accuracy of the model will be evaluated using the area under the receiver operator characteristic curve.

The resulting clinical prediction tool will be independently validated in the cohort with Perth postcodes as described above, and with model will be considered valid if the derivation and validation area under the curve exceeds 0.75.

If we are able to derive a clinical useful prediction tool this will be disseminated through publication in peer reviewed journals and conference presentations and made available to the public through an open-access online calculator. We have experience of developing and disseminating prediction tools e.g. [www.bronchiectasisseverity.com](http://www.bronchiectasisseverity.com)
