# Supplementary material for: Genetic and pharmacological relationship between P-glycoprotein and increased cardiovascular risk associated with clarithromycin prescription: An epidemiological and genomic population-based cohort study in Scotland, UK
Source: PLoS Med. 2020 Nov 23;17(11):e1003372. doi: 10.1371/journal.pmed.1003372 (PMC7682888; doi:10.1371/journal.pmed.1003372)
Supplement: S1 Table — (DOCX) [file pmed.1003372.s003.docx]

S1 Table. Crude Hazard Ratios for Risk of Cardiovascular Hospitalisation at 14 days Associated with Clarithromycin Use versus Amoxicillin in Subgroups.

|  | **Subgroup (Total number of events/prescriptions)** | **Crude Hazard Ratio (95% CI)** | **p value** | **Interaction p value** |
| --- | --- | --- | --- | --- |
| Age | <60 years (157/25,817) | 1.67 (1.16-2.41) | 0.006 | 0.08 |
|  | ≥60 years (2,757/179,410) | 1.19 (1.08-1.30) | <0.001 |  |
| Sex | Male (1,422/90,801) | 1.21 (1.06-1.39) | 0.005 | 0.91 |
|  | Female (1,492/114,426) | 1.23 (1.08-1.39) | 0.002 |  |
| Prior Myocardial Infarction | Yes (253/9,177) | 1.60 (1.18-2.18) | 0.003 | 0.07 |
|  | No (2,661/196,050) | 1.19 (1.08-1.32) | <0.001 |  |
| History of COPD | Yes (560/33,902) | 1.37 (1.14-1.63) | <0.001 | 0.08 |
|  | No (2,354/171,325) | 1.13 (1.01-1.26) | 0.028 |  |
| History of Diabetes | Yes (782/33,487) | 1.24 (1.04-1.49) | 0.015 | 0.74 |
|  | No (2,132/171,740) | 1.20 (1.08-1.34) | <0.001 |  |
| History of Heart Failure | Yes (244/7,544) | 1.37 (1.00-1.89) | 0.052 | 0.45 |
|  | No (2,803/203,127) | 1.21 (1.10-1.33) | <0.001 |  |
| CYP3A4 use | Yes (880/39,530) | 1.21 (1.03-1.42) | 0.018 | 0.47 |
|  | No (2,034/165,697) | 1.16 (1.03-1.30) | 0.013 |  |
| P-gp use | Yes (1,976/93,343) | 1.30 (1.16-1.45) | <0.001 | 0.09 |
|  | No (938/111,884) | 1.09 (0.92-1.29) | 0.31 |  |
| Amiodarone use | Yes (103/2,954) | 1.50 (0.94-2.38) | 0.09 | 0.35 |
|  | No (2,811/202,273) | 1.20 (1.10-1.32) | <0.001 |  |
| Atorvastatin use | Yes (618/27,911) | 1.18 (0.97-1.45) | 0.10 | 0.82 |
|  | No (2,296/177,316) | 1.22 (1.10-1.35) | <0.001 |  |
| Digoxin use | Yes (496/16,707) | 1.31 (1.04-1.66) | 0.022 | 0.58 |
|  | No (2,418/188,520) | 1.22 (1.11-1.35) | <0.001 |  |
| Diltiazem use | Yes (542/17,961) | 1.20 (0.98-1.47) | 0.07 | 0.89 |
|  | No (2,372/187,266) | 1.18 (1.07-1.31) | 0.001 |  |
| Non-dihydropyridine CCB use | Yes (1,105/56,644) | 1.26 (1.08-1.47) | 0.003 | 0.69 |
|  | No (1,809/148,583) | 1.21 (1.08-1.36) | 0.001 |  |
| **Overall Unadjusted (Crude) Hazard Ratio** | **2,914/205,227** | **1.21 (1.11-1.33)** | **<0.001** |  |

COPD – chronic obstructive pulmonary disease; P-gp – P-glycoprotein; CCB – calcium channel blocker; CI – confidence interval
