# Supplementary material for: Genetic and pharmacological relationship between P-glycoprotein and increased cardiovascular risk associated with clarithromycin prescription: An epidemiological and genomic population-based cohort study in Scotland, UK
Source: PLoS Med. 2020 Nov 23;17(11):e1003372. doi: 10.1371/journal.pmed.1003372 (PMC7682888; doi:10.1371/journal.pmed.1003372)
Supplement: S2 Table — CV, cardiovascular. (DOCX) [file pmed.1003372.s004.docx]

S2 Table. Association of AA Genotype (Lowest Genetically-Predicted P-Glycoprotein Levels) with CV Hospitalisation compared to other GG or GA Genotype in Patients Prescribed Clarithromycin

|  | **Crude Hazard Ratio (95% CI)** | **Adjusted Hazard Ratio (95% CI)** | **p Value** |
| --- | --- | --- | --- |
| **rs1045642** |  |  |  |
| 0-14 days | 0.79 (0.49-1.26) | 0.88 (0.55-1.41) | 0.60 |
| 15-30 days | 0.51 (0.28-0.90) | 0.57 (0.32-1.03) | 0.06 |
| 30 days to 1 year | 1.20 (1.03-1.39) | 1.34 (1.15-1.56) | <0.001 |
| **rs1128503** |  |  |  |
| 0-14 days | 0.82 (0.47-1.42) | 0.87 (0.50-1.52) | 0.63 |
| 15-30 days | 0.75 (0.40-1.38) | 0.77 (0.42-1.43) | 0.41 |
| 30 days to 1 year | 1.12 (0.95-1.34) | 1.22 (1.02-1.45) | 0.025 |

CI – confidence interval
