# Supplementary material for: Genetic and pharmacological relationship between P-glycoprotein and increased cardiovascular risk associated with clarithromycin prescription: An epidemiological and genomic population-based cohort study in Scotland, UK
Source: PLoS Med. 2020 Nov 23;17(11):e1003372. doi: 10.1371/journal.pmed.1003372 (PMC7682888; doi:10.1371/journal.pmed.1003372)
Supplement: Attachment — Submitted filename: PLOS Medicine Reviewer Response IM 030720.docx [file pmed.1003372.s005.docx]

**Requests from the editors:**

**1.Title: Please revise the title to avoid implications of causality, and also indicate the population/setting in the title, we suggest: “Genetic and pharmacological relationship between P-Glycoprotein and increased cardiovascular risk associated with clarithromycin prescription: An Epidemiological and Genomic Population-Based Cohort Study in Scotland, UK” or similar.**

We have changed the title as suggested by the editors.

**2. Prospective Analysis Plan: Did your study have a prospective protocol or analysis plan? Please state this (either way) early in the Methods section.**

**a) If a prospective analysis plan (from your funding proposal, IRB or other ethics committee submission, study protocol, or other planning document written before analyzing the data) was used in designing the study, please include the relevant prospectively written document with your revised manuscript as a Supporting Information file to be published alongside your study, and cite it in the Methods section. A legend for this file should be included at the end of your manuscript.**

**b) If no such document exists, please make sure that the Methods section transparently describes when analyses were planned, and when/why any data-driven changes to analyses took place.**

**c) In either case, changes in the analysis—including those made in response to peer review comments—should be identified as such in the Methods section of the paper, with rationale**

As part of our funding proposal we did include a prospective analysis plan for the longitudinal cohort study. We have included this plan as a supplementary appendix.

The initial aim of the study was to use the findings from the cohort study to develop a clinical risk score for prediction of adverse cardiovascular results when using clarithromycin, however, following our finding of potential interaction with P-glycoprotein substrate/inhibitor use, and recognising that this had not been reported previously, we designed a pharmacogenomic study to further interrogate this. The methodology used is as outlined in the manuscript. We did not have a pre-specified plan for the pharmacogenomic section of the study, however, the analysis was informed by the cohort study.

We changed the primary endpoint of the study from the original analysis plan from CV mortality to CV hospitalisation once we had an initial idea of the number of patients who would be included in our analysis and realised we were likely to have limited power. This is described in the methods section (page 9, lines 1-7).

**3. Data Availability Statement: PLOS Medicine requires that the de-identified data underlying the specific results in a published article be made available, without restrictions on access, in a public repository or as Supporting Information at the time of article publication, provided it is legal and ethical to do so. Please see the policy at**

[**http://journals.plos.org/plosmedicine/s/data-availability**](http://journals.plos.org/plosmedicine/s/data-availability)

**and FAQs at**

[**http://journals.plos.org/plosmedicine/s/data-availability#loc-faqs-for-data-policy**](http://journals.plos.org/plosmedicine/s/data-availability#loc-faqs-for-data-policy)

We have now included this verbatim in the manuscript:

*“De-identified data for both the longitudinal cohort and pharmacogenomic parts of the study are held within the Tayside Health Informatics Centre Safehaven (*[*https://www.dundee.ac.uk/hic/hicsafehaven/*](https://www.dundee.ac.uk/hic/hicsafehaven/)*). Applications for access to the GoDARTS dataset can be made via a Project Collaboration Request Form (*[*https://godarts.org/scientific-community/)*](https://godarts.org/scientific-community/))*.” (Data availability statement, page 5 lines 8-12)*

**Additionally, please provide a link or file containing the STATA and R code as requested by reviewer 1.**

We have created a GitHub repository at <https://github.com/ifymordi> which contains a link to the code used in the creation of the datasets and analysis of the study. We have added the following to the data availability statement:

*“Code used in the construction of the dataset and statistical analysis is available at the following link:* [*https://github.com/ifymordi*](https://github.com/ifymordi)*.” (Data availability statement, page 5, lines 12-13)*

**4. Abstract: Please provide some of the relevant summary demographics for the observational cohort study in Scotland. Please include some information on the population and years during which the study took place for the GoDARTS participants.**

We have added this to the abstract (Methods and Findings section) as follows:

*“…a genomic observational cohort study evaluating genotyped patients from the GoDARTS study, a longitudinal cohort study of 18,306 individuals with and without type 2 diabetes recruited between 1989 and 2015.” (page 2 lines 12-13)*

*“The observational cohort study included 48,026 individuals with 205,227 discrete antibiotic prescribing episodes (34,074 clarithromycin – mean age 73 years, 42% male; 171,153 amoxicillin – mean age 74 years, 45% male).” (page 2 lines 21-23)*

*“In the pharmacogenomic study (13,544 individuals with 44,618 discrete prescribing episodes, [37,497 amoxicillin – mean age 63 years, 56% male; 7,121 clarithromycin – mean age 66 years, 47% male])” (page 3 lines 2-4)*

**5. Abstract: Please quantify the main results with both 95% CIs and p values. Where applicable, please include the important dependent variables that are adjusted for in the analyses.**

In the cohort study hazard ratios were adjusted using the inverse probability of treatment weighting as a a covariate, while in the pharmacogenomic study we adjusted for age, sex and history of myocardial infarction and COPD. We have added this as follows:

*“In the observational cohort study we calculated hazard ratios adjusted for likelihood of receiving clarithromycin using inverse proportion of treatment weighting as a covariate, while in the pharmacogenomic study hazard ratios were adjusted for age, sex, history of myocardial infarction and history of COPD.” (page 2 lines 17-20)*

We have also reported both 95% CIs and p values as requested.

**6. Abstract: In the last sentence of the Abstract Methods and Findings section, please describe the main limitation(s) of the study's methodology.**

The main limitation of our study is its observational nature, although this our findings are strengthened by the use of both a traditional observational approach and a pharmacogenomic study. We have included a line on this weakness as requested by the editors as follows:

*“The main limitation of our study is its observational nature, meaning that we are unable to definitively determine causality.” (page 3 lines 9-10)*

**7. Author Summary: At this stage, we ask that you include a short, non-technical Author Summary of your research to make findings accessible to a wide audience that includes both scientists and non-scientists. The Author Summary should immediately follow the Abstract in your revised manuscript. This text is subject to editorial change and should be distinct from the scientific abstract.**

**Please see our author guidelines for more information:** [**https://journals.plos.org/plosmedicine/s/revising-your-manuscript#loc-author-summary**](https://journals.plos.org/plosmedicine/s/revising-your-manuscript#loc-author-summary)

We have included an Author Summary as per the author guidelines as follows (pages 4 and 5):

*“Why was this study done?*

*•	Macrolide antibiotics such as clarithromycin are frequently used for treatment of lower respiratory tract infections.*

*•	Several studies have reported increased cardiovascular risk associated with clarithromycin prescription, including the CLARICOR randomised-controlled trial.*

*•	The mechanism behind this increased risk is unclear, however it would be important to identify patients who might be at higher risk when prescribed clarithromycin in whom alternative antibiotics such as amoxicillin could be used.*

*What did the researchers do and find?*

*•	We conducted an observational study in two parts to examine the association between clarithromycin prescription and cardiovascular outcome.*

*•	First we performed a propensity-weighted observational population cohort analysis comparing clarithromycin vs. amoxicillin prescription and cardiovascular outcome in patients in Tayside, Scotland.*

*•	In this analysis we found that that patients prescribed clarithromycin were significantly more likely to have a cardiovascular hospitalisation at 0-14 days and 30 days to 1 year after prescription than those prescribed amoxicillin.*

*•	This association was modified by concomitant prescription of P-glycoprotein substrates or inhibitors, with individuals who were co-prescribed P-glycoprotein substrates or inhibitors and clarithromycin at particularly high-risk of cardiovascular hospitalisation.*

*•	To explore these findings further, we performed a pharmacogenomic study involving patients with available genotype data from the Genetics of Diabetes Audit and Research Tayside Scotland (GoDARTS) study.*

*•	Using 2 genetic variants associated with P-glycoprotein activity (rs1045642 and rs1128503), we found that patients with the AA genotype, which for both variants is associated with reduced P-glycoprotein activity, had a significantly increased risk of cardiovascular hospitalisation between 30 days and 1 year.*

*What do these findings mean?*

*•	Our findings raise suggest that the observed association between clarithromycin prescription and increased cardiovascular risk could be mediated by P-glycoprotein.*

*•	It is possible that clarithromycin should be avoided in patients taking P-glycoprotein inhibitors or with low genetically-predicted P-glycoprotein activity.”*

**8. Results: Please present p values as p<0.001 where applicable.**

We have been through the manuscript to correct this as requested.

**9. Results: At the bottom of page 9: Please revise the following sentence, as the term "trend" is used to refer to a nonsignificant p value. The term trend should be used only when the test for trend has been conducted. “There was also a non-significant trend to higher likelihood of hospitalisation for MI within the first 14 days of clarithromycin.”**

We have revised the sentence which now reads as follows:

*“A higher proportion of those taking clarithromycin had MI requiring hospitalisation within 14 days, though this difference was not statistically significant.” (page 11, lines 4-5)*

**10. Results: Please present the numerators and denominators when reporting percentages (at least in a table if not in the text); for example, describing the pharmacogenetic cohort: “Individuals prescribed clarithromycin were also more likely to have had a prior MI (13.1% vs. 4.5%, p<0.001) and a history of COPD (29.9% vs. 17.9%, p<0.001).”**

The denominator is the number of prescriptions. We have now reported the numerator and denominator for each variable in the text as requested as follows:

*“Patients who were prescribed clarithromycin were older (66.4 ± 12.4 vs. 63.2 ± 13.2 vs. years, SMD 0.246) and more likely to be male (3,959/7,121 prescriptions - 55.6% vs. 17,549/37,497 - 46.8%, SMD 0.177). Individuals prescribed clarithromycin were also more likely to have had a prior MI (933/7,121 - 13.1% vs. 1687/37,497 - 4.5%, SMD 0.307) and a history of COPD (2,135/7,121 - 29.9% vs. 6,712/37,497 - 17.9%, SMD 0.284). There was no significant difference in the percentage of patients with diabetes (6,472/7,121 - 90.9% vs. 33,822/37,497 - 90.2%, SMD 0.024).”* *(page 11 lines 19-24)*

**11. Results: Please present both the unadjusted and adjusted results where applicable, at least in the tables if not in the text. For example, please present the unadjusted results for the relationship between clarithromycin and CV hospitalization risk described on page 13.**

As requested, we have now presented the unadjusted results (under the column heading “crude hazard ratio”) for both analyses in manuscript Tables 3 and 6.

**12. Results: Page 13: Please clarify this sentence, as it seems like there was also an increased risk at 15-30 days. “After adjustment for age, sex, history of myocardial infarction and history of COPD, clarithromycin prescription was associated with increased risk of CV hospitalisation between 30 days and 1 year (0-14 days: HR 1.23, 95% CI 0.95-1.58, p=0.12; 15-30 days: HR 1.50, 95% CI 1.13-1.99, p=0.005; 30 days-1 year: HR 1.10, 95% CI 1.01-1.19, p=0.031).”**

We thank the editors for noticing this omission. We also added presence of type 2 diabetes to this model. We have clarified the sentence which now reads:

*“In this cohort, irrespective of genotype, clarithromycin prescription was associated with increased risk CV hospitalisation compared to amoxicillin at 15-30 days and 30 days to 1 year (crude hazard ratios: 0-14 days 1.88; 95% CI 1.48-2.38, p<0.001, 15-30 days 2.27; 95% CI 1.74-2.95, p<0.001, 30 days-1 year 1.57; 95% CI 1.45-1.70, p<0.001; adjusted hazard ratios: 0-14 days 1.24; 95% CI 0.96-1.60, p=0.10, 15-30 days 1.50; 95% CI 1.14-1.99, p=0.004, 30 days-1 year 1.10; 95% CI 1.01-1.19, p=0.027).” (page 12 lines 9-14)*

**13. Discussion, page 15: Please temper the following sentence with “To the best of our knowledge” or similar: “Our study is the first to specifically examine a treatment interaction with P-glycoprotein inhibitors and substrates.”**

We have amended as suggested by the editors (page 13, lines 11-12).

**14. Discussion: Please present and organize the Discussion as follows: a short, clear summary of the article's findings; what the study adds to existing research and where and why the results may differ from previous research; strengths and limitations of the study; implications and next steps for research, clinical practice, and/or public policy; one-paragraph conclusion. You touch on implications for clinical practice throughout the discussion, but please consolidate together in a paragraph if possible.**

We have now reformatted the discussion as requested by the editors with sub-headings relating to each section suggested.

**15. Discussion (and throughout): Please replace "Caucasian" with "white" throughout the paper.**

We have changed this as requested.

**16. Table 1: For age, please indicate in the table or legend if you are reporting mean +/- SD, or some other measure here.**

We have added [mean ± SD] to clarify this as follows in this extract of Table 1:

|  | **Clarithromycin** | **Amoxicillin** |
| --- | --- | --- |
| Total Number of Unique Patients | 11,489 | 36,537 |
| Total Number of Prescriptions | 34,074 | 171,153 |
| Age at Prescription (years) [mean ± SD] | 73.3 ± 12.3 | 74.2 ± 13.2 |
| Male | 14,284 (41.9) | 76,597 (44.7) |

**17. Table 2: Please clarify what the values are representing in the legend “Figure in brackets refers to the number of events as a percentage of the total number of prescriptions.” as there are parentheses in the table but no brackets. If the values in parentheses are the percentages of the total number of prescriptions, please place the number of prescriptions in the column headers, for reference. In the legend, please define the abbreviation for ‘MI’.**

Apologies, we did mean figures in parentheses. We have made the requested changes to Table 2 as follows:

Table 2. Clinical Outcomes in the Observational Cohort study (unadjusted counts and percentages).

|  |  | **Clarithromycin**  **(n=34,074)** | **Amoxicillin**  **(n=171,153)** |
| --- | --- | --- | --- |
| **Cardiovascular Hospitalisation** | 0-14 Days | 559 (1.7) | 2355 (1.4) |
|  | 15-30 Days | 431 (1.3) | 2105 (1.2) |
|  | 30 Days-1 Year | 1828 (5.4) | 11321 (6.6) |
| **Hospitalisation for MI** | 0-14 Days | 34 (0.1) | 169 (0.1) |
|  | 15-30 Days | 23 (0.07) | 131 (0.08) |
|  | 30 Days-1 Year | 164 (0.5) | 1076 (0.6) |
| **Cardiovascular Mortality** | 0-14 Days | 73 (0.2) | 532 (0.3) |
|  | 15-30 Days | 69 (0.2) | 558 (0.3) |
|  | 30 Days-1 Year | 508 (1.5) | 3925 (2.3) |
| **All-Cause Mortality** | 0-14 Days | 289 (0.8) | 1601 (1.0) |
|  | 15-30 Days | 277 (0.8) | 1722 (1.0) |
|  | 30 days-1 Year | 1530 (4.5) | 9991 (5.8) |

Figures in parentheses refer to the number of events as a percentage of the total number of prescriptions. MI – myocardial infarction

**18. Table 3: Please present the unadjusted results in addition to the adjusted HRs. Please define abbreviations for MI, CI, and IPTW in the legend.**

We have now presented the unadjusted results as requested.

**19. Figures 1 and 2: Please present the unadjusted results as well. Please specify the variables controlled for in the legend, and define abbreviations for CV, MI, COPD, HF, PGP, CCB, and CI.**

As requested we have now added unadjusted results and defined the abbreviations in full. Results were adjusted for age at prescription, sex, prior history of chronic obstructive pulmonary disease, prior myocardial infarction, history of type 2 diabetes, left ventricular systolic function impairment and all medications listed in Table 1 and this has also been included as footnote to the tables and figures.

Unadjusted results are now presented as Supplementary Table 1.

**20. References: Please place in-text citations in square brackets, like this [1].**

Our citations and references are now formatted using the PLoS Endnote style.

**21. Checklist: Thank you for including the STROBE checklist as Supporting Information. Please revise the checklist, using section and paragraph numbers, rather than page numbers to refer to locations in the manuscript.**

A revised STROBE statement has been submitted as supplementary data.

**Comments from the reviewers:**

**Reviewer #1: The paper examines the effect of possible drug-drug interactions between clarithromycin and P-glycoprotein (P-gp) inhibitors on cardiovascular risk. The authors consider one observational and one pharmacogenomic cohort in a setting that contrasts how prescriptions of clarithromycin and amoxicillin impact future hospitalizations due to cardiovascular events. The results highlight a P-gp-centric molecular mechanism explaining the bioavailability of clarithromycin and suggest the existence of an at-risk population with a genetic variant coding for low P-gp activity.**

**Overall, the paper is well-written and easy to follow. The hypothesis is clearly formulated and placed in the context of published literature. The usage of observational and pharmacogenomic cohorts strengthens the findings, which, if confirmed, may have an immediate impact on the prescription and dosage of clarithromycin in the clinic. However, several aspects of the statistical analysis currently give pause and must be revisited to ensure that the findings and conclusions are robust.**

We thank the reviewer for their overall positive appraisal of our manuscript.

**Since the focus is on drug-drug interactions, the authors need to be more clear about when clarithromycin is considered by itself vs. in combination with P-gp inhibitors. The Methods section states that the study contrasts "all patients over 18 years old who were prescribed clarithromycin (alone or in combination with another antibiotic) over this period, with those prescribed amoxicillin only as a control group", which gives the impression that clarithromycin monotherapy and combination therapy are always considered as a single group. If this is the case, then how do we know that the clinical outcomes reported in Tables 2 and 3 are not confounded by whether clarithromycin was prescribed alone or in combination with a P-gp inhibitor since amoxicillin is always considered as a monotherapy? (In fact, the subsequent analysis reported in Figures 1 and 2 shows that such a confounder is indeed likely.)**

We apologise to the reviewer as we have perhaps not explained this clearly enough.

Clarithromycin may sometimes be prescribed with other antibiotics, including amoxicillin, whereas amoxicillin is often used as a sole antibiotic. Because clarithromycin is often prescribed alongside amoxicillin, we classified individuals prescribed both of these antibiotics at the same time as being in the clarithromycin group rather than in the amoxicillin group. The use of P-gp inhibitors at the same time was not used as part of the inclusion criteria.

We have revised the sentence mentioned by the reviewer to read as follows:

*“Prescribing data between 2004 and 2014 was used to identify all patients over 18 years old who were prescribed clarithromycin (alone or in combination with amoxicillin or another antibiotic) over this period. A control group of individuals prescribed amoxicillin as a sole antibiotic was also identified.” (page 7, lines 20-23)*

**Along the same vein, I am not sure if amoxicillin monotherapy is a proper control for evaluating the effect of prescribing clarithromycin in combination with a P-gp inhibitor. It seems that either 1) amoxicillin should also be paired with a P-gp inhibitor, or 2) the control group should consist of concomitant prescriptions of clarithromycin and "another drug that is not a P-gp inhibitor". Otherwise, one could argue that all the observed differences in clinical outcomes are simply due to the number of prescribed drugs.**

We understand the reviewer’s comment here which is likely a result of the lack of clarity in the original description of the methods, which we have amended as discussed in the comment above. As explained, the main analysis was of clarithromycin vs. amoxicillin, and because clarithromycin is sometimes prescribed with amoxicillin, we classified individuals prescribed both antibiotics (regardless of any other prescription) in the clarithromycin group. Therefore, the concomitant use of P-gp inhibitors was also accounted for in the amoxicillin group, hence our ability to determine the potential interaction in which we then found that the increased CV risk associated with clarithromycin vs. amoxicillin was significantly modified by concomitant P-gp inhibitor/substrate use, with those also prescribed these being at even higher CV risk if prescribed clarithromycin vs. amoxicillin.

We hope that this clarifies the reviewer’s point adequately.

**The pharmacogenomic analysis is well formulated and cleanly executed, other than the possible ramifications of the control group considerations above.**

The pharmacogenomic analysis uses the same structure as the cohort study, and we hope the explanations given above should clarify this for the reviewer.

**All reported p-values need to be adjusted for multiple hypothesis testing. The authors state that "a p value <0.05 was considered statistically significant", but without adjustment this significance threshold implies that approximately one out of every 20 tests may produce a false positive.**

We thank the reviewer for this suggestion. We accept the reviewers point that due to the design of our study, with outcomes assessed at 3 different time points, there are multiple opportunities to achieve a result with a p value of <0.05, which is not actually always appropriate. We have sought further statistical advice from an independent statistician (Dr Mike Lonergan, Senior Statistician and Epidemiologist at the University of Dundee, now acknowledged in the manuscript).

We do feel that because the outcomes overlap and are correlated, straightforward corrections such as a Bonferroni correction might be excessively harsh. It is also important to note that the majority of “significant” results in our study have p<0.001 (and indeed are actually lower than this – see editorial request number 8 where we had to revise up the p values reported from the initial draft where we had provided p values to a lower magnitude), and so even with such a strict correction these results would still be “significant”.

We have now removed the sentence “a p value <0.05 was considered statistically significant”. We have also removed the bold highlighting p values <0.05 in the tables, and have simply just reported the p values (and corresponding confidence intervals), which can be interpreted within the context of the study.

**Minor comments:**

**1. The statement "To the best of our knowledge, there have been no studies evaluating the association between clarithromycin use, CV risk and P-gp." is a bit too strong. The study by Wessler and colleagues (which is cited in the manuscript) has certainly considered clarithromycin in the context of CV risk and P-gp, as part of a larger study.**

We assume the reviewer is referring to the paper which we have cited as reference number 18:

Wessler JD, Grip LT, Mendell J, Giugliano RP. The P-glycoprotein transport system and cardiovascular drugs. J Am Coll Cardiol. 2013 Jun 25;61(25):2495-502.

This is a comprehensive review article which outlines the importance of P-glycoprotein in cardiovascular pharmacotherapy. In our original statement, we meant that we had not identified any original research studies specifically evaluating the association, rather than review articles. On further review, we are still not aware of any original research publications in this area, although if the reviewer knows of any that we have not identified we would be happy to include it.

We accept that our statement might be a little strong however and have revised as follows:

*“To the best of our knowledge, there have been no original research studies evaluating whether the association between clarithromycin use and CV risk is modulated via P-gp”(page 6, lines 19-21)*

**2. The STATA and R code used for the analysis should be released alongside the manuscript (e.g., as a GitHub repository) to increase reproducibility.**

We have created a GitHub repository at <https://github.com/ifymordi> which contains a link to the code used in the creation of the datasets and analysis of the study.

**3. What is the purpose of reporting a p value for "Total Number of Unique Patients" and "Total Number of Prescriptions" in Table 1? Is the goal to demonstrate that overall one drug is prescribed significantly more often than the other? I don't think that's very surprising...**

We agree that this is unnecessary. We have removed the p values as suggested.

**Reviewer #2: This is an interesting analysis of an important but poorly understood phenomenon, the long-term cardiovascular risks following use of clarithromycin. I believe it would be a useful addition to the literature but there are several ways the manuscript could be improved and there are also some items that need to be clarified.**

We thank the reviewer for their appraisal of our manuscript.

**My major concern with the longitudinal cohort study is the possibility of residual confounding; in particular, indication was not accounted for, a limitation which should be noted in the Discussion. The only two significant HRs are modest in magnitude (1.31, 1.13) and so are in the range that could be the result of confounding. Were the p-values in Table 3 adjusted for multiple comparisons? Also, when you discuss the impact of weighting in the Discussion you note that before weighting the risk of CV events was higher with amoxicillin. It wasn't clear what result that refers to; was it the 15-30 day window?**

We agree with the reviewer that although most prescriptions for both antibiotics (and clarithromycin in particular) are for lower respiratory tract infections, there could be some confounding by indication and we could not adjust for this in our analysis. We have added comment on this in the limitations as suggested:

*“We could also not robustly ascertain prescribing indication and adjust for this, although the majority of prescriptions are likely to have been for suspected lower respiratory tract infection.” (page 15, lines 7-8)*

The discussion around weighting is a comment on the crude outcomes reported in Table 2. In several of the outcomes at various timepoints the number of events as a percentage of the number of prescriptions is higher in the amoxicillin prescribed group than clarithromycin:

Table 2. Clinical Outcomes in the Observational Cohort study (unadjusted counts and percentages).

|  |  | **Clarithromycin**  (n=34,074) | **Amoxicillin**  (n=171,153) |
| --- | --- | --- | --- |
| **Cardiovascular Hospitalisation** | 0-14 Days | 559 (1.7) | 2355 (1.4) |
|  | 15-30 Days | 431 (1.3) | 2105 (1.2) |
|  | 30 Days-1 Year | 1828 (5.4) | 11321 (6.6) |
| **Hospitalisation for MI** | 0-14 Days | 34 (0.1) | 169 (0.1) |
|  | 15-30 Days | 23 (0.07) | 131 (0.08) |
|  | 30 Days-1 Year | 164 (0.5) | 1076 (0.6) |
| **Cardiovascular Mortality** | 0-14 Days | 73 (0.2) | 532 (0.3) |
|  | 15-30 Days | 69 (0.2) | 558 (0.3) |
|  | 30 Days-1 Year | 508 (1.5) | 3925 (2.3) |
| **All-Cause Mortality** | 0-14 Days | 289 (0.8) | 1601 (1.0) |
|  | 15-30 Days | 277 (0.8) | 1722 (1.0) |
|  | 30 days-1 Year | 1530 (4.5) | 9991 (5.8) |

The discussion is a comment on the fact that this apparent discrepancy may be due to the fact that clinicians may avoid clarithromycin in patients who they perceive to be at lower risk (e.g. younger), and once adjustment is made for this (e.g. adjusting for IPTW) then the opposite association is seen, as in our study and others e.g. Paul et al. 2007 (ref 36).

We have revised this segment of the discussion to clarify:

*“There was a higher crude number of events in the amoxicillin group than those prescribed clarithromycin (Table 2), however, after propensity-score adjustment, clarithromycin prescription was associated with worse outcome.” (page 13, lines 18-21)*

Regarding adjustment for multiple comparisons, we have sought further statistical advice from an independent statistician (Dr Mike Lonergan, Senior Statistician and Epidemiologist at the University of Dundee, now acknowledged in the manuscript).

We do feel that because the outcomes overlap and are correlated, straightforward corrections such as a Bonferroni correction might be excessively harsh. It is also important to note that the majority of “significant” results in our study have p<0.001 (and indeed are actually lower than this – see editorial request number 8 where we had to revise up the p values reported from the initial draft where we had provided p values to a lower magnitude), and so even with such a strict correction these results would still be “significant”.

Accepting that during the study there may be multiple opportunities to achieve a p value <0.05, we have now removed the sentence “a p value <0.05 was considered statistically significant”. We have also removed the bold highlighting p values <0.05 in the tables, and have simply just reported the p values (and corresponding confidence intervals), which can be interpreted within the context of the study.

**The most persuasive evidence for a role of P-gp comes from the pharmacogenomic study and the interaction seen with genetically determined low P-gp activity, which as point out is unlikely to be due to confounding. I think you should seriously consider revising the paper to highlight the pharmacogenomic study rather than the longitudinal cohort study, perhaps by switching the order in which the studies are described in the manuscript. Have you compared the risk within clarithromycin-exposed patients alone; i.e., clarithromycin users with GG/GA genotype versus clarithromycin users with AA genotype? This comparison, if significant, would be even more persuasive, but may not be adequately powered in your sample, however.**

We agree with the reviewer that the pharmacogenomic study is a key strength and novelty of our manuscript and thank them for their comment. We do feel that the manuscript does flow better with the cohort study results presented first however. This does also reflect the order in which the hypotheses were generated and analyses were conducted too. We would however consider changing the manuscript structure if the editor felt this would be a better approach.

Regarding the comparison in clarithromycin-exposed patients only; while we understand the concept the reviewer is suggesting, we feel that the way our analysis is presented is perhaps slightly more reflective of the clinical utility of a pharmacogenomic approach i.e. the independent variable is the patient’s genotype which is unchanged regardless of prescription, and therefore the risk assessment of interest is the relative risk of prescribing clarithromycin vs. amoxicillin, which we have presented in Table 6.

If we understand correctly, the reviewer is suggesting that we look at the relative risk of low-Pgp genotype vs. high Pgp genotype individuals if prescribed clarithromycin, essentially switching the independent and dependent variables. This would be a slightly different study design from the cohort study - the analogue of this in the cohort study would be to determine the relative risk of concomitant Pgp use in only the patients given clarithromycin, rather than the relative risk of clarithromycin vs. amoxicillin, which is a key part of the clinical question as we suggest. We have tried to keep the two studies harmonious in their design. The relative risk of each genotype in clarithromycin use can be inferred from table 6, and of course, the interaction p value (which is the key statistic showing a gene-clarithromycin interaction) is the same whichever direction the question is looked at (i.e. risk of clarithromycin vs. amoxicillin stratified by genotype or risk of genotype stratified by clarithromycin exposure).

We have kept the presentation of our analysis as it stands however if the reviewer and editors feel that it is important to show this alternative analysis then we would be happy to include this as a supplementary table.

**Regarding Table 1 (observational longitudinal cohort study): Use of a t-test or chi-square test to assess baseline differences between groups results in many statistically significant differences which are small in magnitude, but statistically significant because of the large sample size. A better approach would be to compare the groups with standardized differences, for which a difference of 0.1 or more is considered consequential. After IPTW, the standardized differences can be compared again to determine if the weighting successfully balanced the baseline covariates. Your manuscript does not address the issue of whether the IPTW succeeded in balancing the baseline covariates. See Mamdani et al., at** [**https://www.bmj.com/content/330/7497/60.long**](https://www.bmj.com/content/330/7497/60.long)

We thank the reviewer for their suggestion and directing us to this reference. We agree that use of standardised differences would be more appropriate given the cohort size in our study, and we have calculated this as requested and included this in a revised Table 1 rather than p values. We have added this to the methods as follows:

*“Standardised mean differences between the clarithromycin and amoxicillin groups are reported.” (page 9, lines 14-16)*

Notably only 4 variables (history of COPD, beta-blocker use, loop diuretic use and use of CYP3A4 inhibitor/substrates) had a standardised difference >0.1 between the two groups. This has be commented on in the results:

*“Overall, individuals prescribed clarithromycin more likely to have COPD and be prescribed loop diuretics and CYP3A4 inhibitors or substrates, and were less likely to be taking beta-blockers (SMD >0.1).” (page 10, lines 12-14)*

We have perhaps not made it clear within the manuscript, however it is important to note that we performed a propensity-weighted analysis, not a propensity-score matched analysis. We generated weighting using inverse probability of treatment weighting (in this case likelihood of receiving clarithromycin rather than amoxicillin) which performs as well as propensity score matching and allows retention of all participants within the dataset as described by Austin (Stat Med 2013).

*“Adjusted hazard ratios in the observational study are reported adjusting for the inverse probability treatment weighting (IPTW), using the propensity score for likelihood of prescription of clarithromycin based on baseline variables reported in Table 1” (page 9, lines 19-21)*

Given this, we did not have a “post-matching” dataset as no matching was performed.

**In the observational longitudinal cohort study, it is puzzling why the mean age is so high (about 73 years for clarithromycin users), if any patient over 18 years of age was included. Can you please comment?**

We agree with the reviewer that it is notable that the mean age is 73 (although it is also 74 for the amoxicillin group) and that this does differ from some other published studies. It is likely this simply reflects the increased likelihood of older patients having lower respiratory tract infections requiring medical attention and antibiotic prescription. Our cohort is also unselected in that it will have included patients with infective exacerbations of COPD and pneumonia, and thus may be a slightly older population than other studies. Nevertheless, the mean age of our cohort is not dissimilar from that reported by Schembri et al (BMJ 2013) (median age 72 in COPD cohort, 66 in pneumonia cohort), which also reported data from Scotland.

It is also notable that the mean age is 10 years lower in the pharmacogenomic study. We speculate that this perhaps relates to the well-reported phenomenon of older individuals being less likely to be included in clinical trials, and so an electronic health record study with broad inclusion criteria such as ours might have a higher mean age.

**Study endpoints: Was ICD-10 in use in Scotland during the entire study period (2004-2014)?**

We thank the reviewer for raising this point.

Scotland adopted ICD-10 in 1996, as documented on the “Public Health Scotland” website - <https://www.isdscotland.org/Products-and-Services/Terminology-Services/Coding-and-Terminology-Systems/>.

**Minor comments**

**Table 2: suggest showing total number of patients per group in the header row**

We have amended table 2 as suggested by the reviewer.

|  |  | **Clarithromycin**  **(n=33,759)** | **Amoxicillin**  **(n=169,002)** |
| --- | --- | --- | --- |
| **Cardiovascular Hospitalisation** | 0-14 Days | 559 (1.7) | 2355 (1.4) |
|  | 15-30 Days | 431 (1.3) | 2105 (1.2) |
|  | 30 Days-1 Year | 1828 (5.4) | 11321 (6.6) |
| **Hospitalisation for MI** | 0-14 Days | 34 (0.1) | 169 (0.1) |
|  | 15-30 Days | 23 (0.07) | 131 (0.08) |
|  | 30 Days-1 Year | 164 (0.5) | 1076 (0.6) |
| **Cardiovascular Mortality** | 0-14 Days | 73 (0.2) | 532 (0.3) |
|  | 15-30 Days | 69 (0.2) | 558 (0.3) |
|  | 30 Days-1 Year | 508 (1.5) | 3925 (2.3) |
| **All-Cause Mortality** | 0-14 Days | 289 (0.8) | 1601 (1.0) |
|  | 15-30 Days | 277 (0.8) | 1722 (1.0) |
|  | 30 days-1 Year | 1530 (4.5) | 9991 (5.8) |

**Figure 2: is the risk window here also 14 days? Are all these drugs P-gp substrates? If so, why does there only appear to be an interaction with dihydropyridine CCBs and maybe amiodarone?**

Yes, the reviewer is correct that this figure refers to 14-day CV hospitalisation and we have amended the title to reflect this.

Based on Wessler et al (JACC 2013, reference 18), amiodarone and dihydropyridine CCBs are inhibitors of Pgp, while digoxin is a substrate. Diltiazem and atorvastatin are both inhibitors and substrates. There may be a relation between the amount of inhibition (vs. being a substrate) and the effect on clarithromycin, however this is just speculation on our part and unfortunately we are underpowered to draw any definitive conclusions on this. This would certainly be a very interesting area for further mechanistic work and we thank the reviewer for raising this point. We have touched on this in the “Next steps” section of the discussion:

*“Mechanistic studies evaluating the pathophysiology of P-glycoprotein-associated gene and drug interactions in detail would further our understanding and inform future clinical practice.” (page 16, lines 4-5)*

**Table 4: the percentages for rs1045642 don't sum properly (total = 110.5%)**

We thank the reviewer for identifying this typographical error. This should read 22.1% (1575/7121) and has been amended in the manuscript.

**Discussion: You state that amoxicillin and tetracyclines are non-inferior to macrolides for respiratory infections. Some have argued that macrolides reduce mortality from community acquired pneumonia, however. See for example Asadi et al.,** [**https://academic.oup.com/cid/article/55/3/371/613119**](https://academic.oup.com/cid/article/55/3/371/613119)

We thank the reviewer for drawing our attention to this meta-analysis by Asadi et al published in 2012. Although the overall meta-analysis including observational studies did find a 22% reduction in mortalty with macrolides, it is notable that in the meta-analysed results including only randomised trials or only including studies in whom patients were given guideline-recommended treatment, there was no mortality benefit for macrolides, and in fact macrolides were non-significantly associated with increased mortality compared to the comparator.

We also note that the most recent 2019 American Thoracic Society Community Acquired Pneumonia Guidelines recommend amoxicillin and doxycycline as initial treatment for community-acquired pneumonia, and “did not give a strong recommendation for routine use of a macrolide antibiotic as monotherapy for outpatient CAP, even in patients without comorbidities”, although they do suggest they can be used if macrolide resistance is low and there are contraindications to alternative therapies.

We do accept however that there is a place for macrolide antibiotics. We have rephrased the section of the manuscript to reflect the reviewer’s comment as follows:

*“Macrolides are primarily used to treat respiratory infections, however alternatives such as amoxicillin or tetracyclines are not associated with increased CV risk, not metabolised through P-glycoprotein, and have been shown in randomised trials to be non-inferior to treatment regimens including macrolides [44, 45]. The most recent American Thoracic Society guidelines for community acquired pneumonia which recommend the use of macrolide monotherapy only in areas where macrolide resistance is low and in patients in whom alternative antibiotics are contraindicated [46].” (page 15, lines 15-20)*

We have also cited the paper by Asadi et al as reference number 45.

**Final sentence: It is probably more appropriate to speak of drugs that are substrates for P-gp rather than "metabolized" by P-gp.**

We agree with the reviewer that this is more appropriate. We have amended as follows:

*“There appears to be an effect modification via P-glycoprotein, with a particularly increased risk of adverse CV events with clarithromycin in patients also taking drugs that are P-glycoprotein substrates or those with lower genetically-predicted levels of P-glycoprotein activity.” (page 16, lines 8-11)*
