# Supplementary material for: Genetic and pharmacological relationship between P-glycoprotein and increased cardiovascular risk associated with clarithromycin prescription: An epidemiological and genomic population-based cohort study in Scotland, UK
Source: PLoS Med. 2020 Nov 23;17(11):e1003372. doi: 10.1371/journal.pmed.1003372 (PMC7682888; doi:10.1371/journal.pmed.1003372)
Supplement: Attachment — Submitted filename: Editor Response 210820.docx [file pmed.1003372.s006.docx]

**Requests from Editors:**

**1. Data availability statement: As mentioned by reviewer 1, please include the GitHub link specific for your dataset used in this study.**

This has been done as requested by reviewer 1. The direct link is <https://github.com/ifymordi/Clarithromycin>.

**2. Abstract: Background: First sentence: Please change the word “and” to “with”**

Amended.

**3. Abstract: Background: Please revise to temper this claim, we suggest: “To the best of our knowledge, no studies have examined whether this association might be mediated via P-glycoprotein (P-gp), a major pathway for clarithromycin metabolism.**

Amended as suggested.

**4. Abstract: Methods and Findings: There appears to be a missing p value for the pharmacognetic report of the AA allele associated with lower p-gp activity (rs1045642 AA: HR 1.39, 95% CI 1.20-1.60)**

P value (<0.001) added.

**5. Abstract: Conclusions: We suggest revising to: “In this study, we observed that that increased risk of CV events with clarithromycin compared to amoxicillin were associated with an interaction with P-glycoprotein.” or similar to temper the causal implications.**

As suggested, we have amended to:

*“In this study, we observed that the increased risk of CV events with clarithromycin compared to amoxicillin was associated with an interaction with P-glycoprotein.”*

**6. Author summary: What did the researchers do and find?: We suggest combining the third and fourth bullet points as follows:**

**“-In this analysis we found that that patients prescribed clarithromycin were significantly more likely to have a cardiovascular hospitalisation at 0-14 days and 30 days to 1 year after prescription than those prescribed amoxicillin, and that individuals who were co-prescribed P-glycoprotein substrates or inhibitors and clarithromycin had significantly higher risk of cardiovascular hospitalisation.”**

Amended as suggested.

**7. Author summary: What do these findings mean: We suggest revising the second point as follows:**

**“These results suggest implications for clarithromycin use patients taking P-glycoprotein inhibitors or with low genetically-predicted P-glycoprotein activity.” or similar.**

Amended as follows:

*“These results may have implications for clarithromycin use in patients taking P-glycoprotein inhibitors or with low genetically-predicted P-glycoprotein activity.”*

**8. Data availability statement: Please remove this section from the body of the manuscript and ensure it is entered accurately (with updated GitHub information) where appropriate in the manuscript submission system form.**

We have done this as suggested. We have also updated the Github information as suggested by reviewer 1.

**9. Methods: Page 7: Regarding your prospective analysis plan, thank you for noting in your response to comments that “We did not have a pre-specified plan for the pharmacogenomic section of the study, however, the analysis was informed by the cohort study.” Please include a statement such as this in Methods.**

We have included this in the methods as follows:

*“We did not have a pre-specified analysis plan for the pharmacogenomic study, however, the analysis was informed by the observational cohort study.” (page 8 lines 5-6)*

**10. Methods: Study Endpoints: First sentence, and throughout: Please consistently use the abbreviation “CV” for cardiovascular, defining the abbreviation at first use in the text and using the abbreviation throughout, for the sake of consistency: “The primary endpoint for both studies was CV hospitalisation. In our 1 initial funding proposal we planned to evaluate cardiovascular mortality as the primary endpoint…”**

Amended as suggested.

**11. Methods: Please add the following statement, or similar, to the Methods: "This study is reported as per the Strengthening the Reporting of Observational Studies in Epidemiology (STROBE) guideline (S1 Checklist)."**

This statement has been added verbatim on page 10 lines 7-8.

**12. Results Page 11: Clinical outcomes for Observational Cohort Study: Please provide the result with 95% CIs and p values here: “A higher proportion of those taking clarithromycin had MI requiring hospitalisation within 14 days, though this difference was not statistically significant.”**

We have included this as requested:

*“A higher proportion of those taking clarithromycin had MI requiring hospitalisation within 14 days, though this difference was not statistically significant (adjusted HR 1.37; 95% CI 0.89-2.11, p=0.12).”*

**13. Results: Page 11: Please also present the HR and 95% CIs for the interactions with P-gp inhibitors at 30 days and 1 year to accompany these p values? “interaction was not seen at 30 days or 1 year (interaction p values 0.74 and 0.53 respectively).”**

We have added this as follows:

*“This interaction was not seen at 15-30 days or 30 days to 1 year (15-30 days: P-gp – HR 0.93; 95% CI 0.49-1.76; no P-gp – HR 0.98; 95% CI 0.56-1.71, interaction p value 0.74; 30 days-1 year: P-gp – HR 0.95; 95% CI 0.73-1.25; no P-gp – HR 0.99; 0.77-1.28, interaction p value 0.53).”*

**14. Discussion: Please start the discussion with 1-2 sentences briefly summarizing what was done in the study.**

We have added this as suggested:

*“We performed a cohort study in two parts, combining a traditional epidemiological approach with a pharmacogenomic study to evaluate the association of the use of the macrolide antibiotic clarithromycin with CV risk.”*

**15. Discussion: Page 13: Please revise to: “These results may suggest that, at least in part, the association of clarithromycin with increased CV risk may be modified via P-glycoprotein and particular caution may be needed in prescribing clarithromycin in individuals taking P-gp inhibitors” or similar**

We have changed as follows:

*“These results may suggest that, at least in part, the association of clarithromycin with increased CV risk may be modified via P-glycoprotein and particular caution may be needed when prescribing clarithromycin in individuals taking P-gp inhibitors.”*

**16. Discussion: Page 13: Please revise to: “This has been previously reported and is because patients prescribed amoxicillin alone tend to be older and more unwell, hence methods such as propensity-score weighting are required to account for this [37], and this is likely to contribute to the non-significant unadjusted increase in mortality seen with amoxicillin in our study” or similar.**

Amended verbatim.

**17. Discussion: page 14: Please change “was” to “is” in the following sentence: “With these proposed mechanisms, any pathway by which metabolism of clarithromycin was impaired might lead to increased CV risk.”**

Amended.

**18. Discussion: page 15: Please replace the term “compliance” with “adherence” where it is used to refer to treatment adherence.**

Amended as suggested.

**19. Discussion: Page 15: Please remove the word “which” to clarify: “The most recent American Thoracic Society guidelines for community acquired pneumonia recommend the use of macrolide monotherapy only in areas where macrolide resistance is low and in patients in whom alternative antibiotics are contraindicated [46].”**

Deleted as requested.

**20. Sections: Declaration of Interests, Funding, Role of the Funding Source: Please remove these sections from the main text of the manuscript, and ensure the information is accurately entered into the relevant locations within the manuscript submission system.**

Removed as requested.

**21. Table 1: Please indicate in the table or legend that the values represent numbers and percentages.**

Added as a footnote.

**22. Table 3: Please define the abbreviation “CI” in the legend.**

Added.

**23. Table 6: Please also define GA/GG in the legend. Please indicate in the column headers that the hazard ratios are followed by the 95% CIs.**

Added as follows:

*“AA – lowest genetically-predicted P-glycoprotein levels; GG – highest genetically-predicted P-glycoprotein levels; GA – intermediate genotype.”*

**24. Figure 1 and Figure 2: Please also define abbreviation for CV in the legend.**

Added.

**25. S1 Table: Please define abbreviations for pgp, CCB, and CI in the legend.**

Added.

**26. Supplementary File 2: Analysis Plan: Thank you for including your analysis plan. If you have a dated version of this plan, please update accordingly.**

As requested the date has been added to the text.

**Comments from Reviewers:**

**Reviewer #1: Overall, I am satisfied with the revisions submitted by the authors and have no other concerns. In particular, I thank the authors for clarifying the nuances of clarithromycin prescription. With the new description, I no longer have reservations about the control group. Very minor comments are below.**

We thank the reviewer for their comments which have certainly improved the manuscript.

**I agree with the authors that the Bonferroni correction is too stringent and would generally recommend something like the recently-proposed harmonic mean p-value (PMID: 30610179), which is able to handle groups of dependent tests without explicit access to what the dependency structure is. However, I think that reporting raw p-values without placing an artificial "significance" threshold is also a reasonable strategy, albeit one that softens the main message.**

**Minor note regarding GitHub organization: The GitHub link in the manuscript (**[**https://github.com/ifymordi**](https://github.com/ifymordi)**) refers to a user, not a repository. As this user (I hope) publishes additional code in future studies, it will become increasingly difficult to find the code associated with the current clarithromycin study by following the link in the paper. Consider renaming your "Research" repository to "Clarithromycin" and updating the link to github.com/ifymordi/Clarithromycin. It also seems that all code is in a branch that is not immediately visible to a user. Consider merging this branch into master, since that's what readers will see when they first follow your link.**

We thank the reviewer for their constructive comment. As suggested, we have revised the link to <https://github.com/ifymordi/Clarithromycin>, which now provides a direct link to the code which is now the default master.

**Reviewer #2: I appreciate the opportunity to review the revised manuscript. I thank the authors for their careful and complete consideration of my comments. The manuscript is much improved and while I have a couple of suggestions I don't feel that any further changes are mandatory. However, for the authors' consideration, I will provide some further thoughts regarding two of my previous comments.**

We thank the reviewer for their constructive comments which we agree have substantially improved the manuscript.

**1. Standardized differences--I would point out that it is possible to calculate standardized differences in baseline characteristics on the weighted population, just as you have now done for before IPTW. This is similar to calculating standardized differences on a propensity-score matched sample both before and after matching. If you show that the post-IPTW standardized differences are minimal (<0.1) that would strengthen the findings of the study.**

**2. Regarding my suggestion for a within-clarithromycin analysis of risk by P-gp genotype: I take the point that the test for the treatment X genotype interaction essentially controls for the effect of amoxicillin. However, I wonder if a within-clarithromycin analysis might provide a more straightforward demonstration of this effect, although one would have to assume that P-gp genotype varies randomly among clarithromycin users. This is just a suggestion for the authors as the existing analysis does address the effect of genotype.**

We have performed this analysis as suggested by the reviewer. As expected, when restricting the analysis only to individuals prescribed clarithromycin, this shows that the AA genotype is associated with increased likelihood of CV hospitalisation between 30 days and 1 year, outlined in the table below:

Association of AA Genotype (Lowest Genetically-Predicted P-Glycoprotein Levels) with CV Hospitalisation compared to other GG or GA Genotype in Patients Prescribed Clarithromycin

|  | Crude Hazard Ratio (95% CI) | Adjusted Hazard Ratio (95% CI) | p Value |
| --- | --- | --- | --- |
| rs1045642 | | | |
| 0-14 days | 0.79 (0.49-1.26) | 0.88 (0.55-1.41) | 0.60 |
| 15-30 days | 0.51 (0.28-0.90) | 0.57 (0.32-1.03) | 0.06 |
| 30 days to 1 year | 1.20 (1.03-1.39) | 1.34 (1.15-1.56) | <0.001 |
|  |  |  |  |
| rs1128503 | | | |
| 0-14 days | 0.82 (0.47-1.42) | 0.87 (0.50-1.52) | 0.63 |
| 15-30 days | 0.75 (0.40-1.38) | 0.77 (0.42-1.43) | 0.41 |
| 30 days to 1 year | 1.12 (0.95-1.34) | 1.22 (1.02-1.45) | 0.025 |

This table has now been included in the Supplementary Table 2, and included in the results section as follows:

*“Restricting the analysis only to those individuals prescribed clarithromycin, for both the rs1045642 and rs1128503 genetic variants, individuals with the AA genotype were more likely to have a CV hospitalisation between 30 days and 1 year than those with the GA or GG genotypes (rs1045642: HR 1.34, 95% CI 1.15-1.56, p<0.001; rs1128503: HR 1.22, 95% CI 1.02-1.45, p=0.025) (S2 Table).”*
